# Free Medical Schools

**Published:** 1850-09

**Authors:** 


### ARTICLE II.

### FREE MEDICAL SCHOOLS.

In a former number of the Journal, we promised to have something further to say in reference to the question of free medical teaching. Since that promise, we have given several articles from the pen of Prof. Davis, defending his position against the criticisms of some of our contemporaries, and in the mean time we have been taking notes of the arguments, and observing the influences exerted by the discusssion.

At one time the idea of cheapening medical education was scouted as a thing not to be tolerated. And so long as college fees were only reduced by competition between rival medical schools underbidding each other, it is true, they deserved no credit for the good that was accomplished, and their motives attracted the attention of the profession, instead of the propriety and influence of the reduction. But since the proposition to bring medical instruction within the reach of all those who enter the profession has been discussed as an abstract principle, and a number of the ablest men in the profession have advocated its propriety, the odium that once attached to the idea has vanished, and the horror excited in the minds of some professors by the proposition to "cheapen medical education," has been followed by a very encouraging diminution in the price of their own tickets. Instance—the Medical College of Ohio in which the senior editor of the *Western Lancet* is a Professor, of whose fears for the respectability of the profession, if medical education should be cheapened, our readers have already heard, has actually reduced the price of the tickets of its professors from fifteen to twelve dollars each!! As showing still more plainly the tendency in the same quarter, we observe the *Lancet* says, in noticing the Circular of the Medical College of Evansville, that *ten* dollars a ticket is respectable!! Another—the Indiana Central Medical College has added another chair, without increasing the aggregate amount of its tickets, so that each professor now receives less than nine dollars a ticket, against ten dollars last year.

Even those who proposed, last Winter, to have the Faculty of Rush Medical College condemned by the American Medical Association, for taking the lead in the great reform of cheapening medical education, and others who proposed that the Association should take action upon the subject, must have materially changed their minds in reference to it, for notwithstanding one of the most eloquent and powerful addresses before the late meeting of that honorable body, by Prof. Davis, advocating the principle of cheap medical teaching, no action was proposed by any other member of that body in reference to it. And instead of condemning the Faculty, one of its members was elected a Vice-President of the Association.

Although in all propositions for advancement in any department of the policy of the country, there will be found "Old Hunkers," whose fixed and stereotyped notions, or whose interests, are disturbed by them, and who consequently condemn them, there is to be found, in the signs of the times, abundant evidence that this one, for rendering instruction to students of medicine more accessible is soon to become the general favorite of the medical profession. E.

